# Filing Teeth

**Published:** 1848-04

**Authors:** 

**Affiliations:** Cincinnati Editor


### FILING TEETH.

BY CINCINNATI EDITOR.

There is no operation on the Teeth which has been so much abused, and which has consequently arrayed against it so much prejudice and opposition. We are satisfied that this prejudice is the result of the most shameful ignorance as to the cause of decay and the manner in which this operation should be performed. It is within our recollection when a large majority of those operating on the Teeth, at least in the West—(we will not say dentists, for they were generally silversmiths, shoemakers, tinkers, coblers, &c., &c.,)—were in the constant habit of recommending the use of the file for the separation of the Teeth. Alledging that the pressure was thereby relieved and decay prevented, with these operators it was just as important to thus separate sound teeth as those decaying.

"An ounce of preventive," say they, "is better than a pound of cure." But who would take physic when in perfect health for fear of being sick? A man who would thus yield to fear is half sick already. But in this case we have no evidence that the teeth will decay;—true we take it for granted, from observation, that teeth much crowded are thus favorably arranged for an attack of disease—yet we often see teeth as much crowded as well can be, remain sound for life—favoring more a disease of the gums than teeth. That pressure alone will induce decay, we think wants evidence.

But let us examine this subject with a little care. We find caries to be a disease induced, we have no doubt, by a local chemical agent, decomposing first the enamel, then more rapidly the dentine, thus forming a cavity, the form and locality of which is favorable for the retention of foreign matter—this undergoing decomposition forms an agent directly at hand by which to carry on the disease. Supplying fuel daily for the further progress of the decay, aided, we have no doubt, very much by the condition of the stomach, and for the time, vitiated secretions of the mouth. We stop not now to prove this position, we believe it is generally admitted. We wish merely to draw attention to the nature of dental caries, before we speak particularly of the remedy proposed to arrest or cure the disease.

Let us now examine the manner in which this operation (filing the teeth,) has been heretofore generally performed. To relieve the pressure, the operator takes a file made for that purpose, and passes it between the teeth, generally removing a portion, or all the enamel from each. This is done extending as far back as the molars, and for the time, pressure is relieved. We are now supposing the teeth unaffected by disease. Observation teaches us that teeth thus separated will approximate. The arch of the jaw is either diminished or some of the spaces are widened while others are closed up, so that the filed surfaces generally left rough by the file comes in contact. We have now presented a case which was of common occurrence a few years back, and we fear still often occurs—and where, instead of a benefit, an injury has been done.

And for this simple reason, the natural protection of the bone has been removed, and a larger and flatter surface comes in contact than before—and thus situated, more liable to be acted upon by chemical agents than before. It is needless, however, to point out the impropriety of separating sound teeth—and did not the cupidity and avarice of some find willing dupes in the credulity of others, it would be unnecessary to allude to it—reason alone points out a far better course: which is, keep the approximal edges well cleaned by forcing between them floss silk, &c.

This same operation is, however, often performed when the teeth are decaying, and done in the same manner and with the same object, viz: the removal of pressure—supposed to be the cause of decay; but as before pressure is only temporarily relieved, the disease is not at all removed,—and indeed no effort appears to have been made for that purpose. Such operations cannot be too strongly condemned, and when we view their frequency we need not be astonished at the prejudice against the use of the file. We take it for granted that the healthy condition of the teeth cannot be improved by the efforts or the skill of man—excepting, so far, indeed, as he may be the means of making them assume a proper position in the arch of the mouth. We take it equally for granted that, where disease has commenced, it is the duty of the dentist to combat and overcome. We would not wish to be understood as proscribing efforts to prevent disease—remedies which will not effect the healthy condition of the parts, those for instance which will neutralize the acids of the mouth, are proper, and we believe that more may be done by regulating the diet of our patients, and method of cleaning their teeth, in effectually preserving these organs than by any other course we can adopt. The position of teeth should be regulated by the dentist at the proper time:—this is easily effected, but should not be done by the use of the file. What is very improper to be done in a healthy condition of things is rendered necessary and proper when diseased. This is strikingly exemplified in the decay of teeth. We find, for instance, the approximal edges of the incissors cuspidati and bicuspides decaying. The enamel is decomposed, and the agent which has effected this has reached the bone, and on this softer substance is acting more rapidly; its action has formed a cavity which admits the fluids of the mouth—retains that which decomposes the tooth—and also particles of food, which may in this condition chemically change their property and hasten the disease.

Here, then, is a condition of things, which, if left alone, must ultimately destroy the tooth. What, then, must be done? One of two things: 1st, the cavity must be filled with a substance capable of resisting any chemical agent that may be introduced or formed in the mouth; or 2d, the disease must be removed. Experience tells us that dead matter, even in the bony structure, is antagonistical to the healthy living tissue. Often the disease has not reached a depth sufficient to retain a filling—often, indeed, the mere separation of the teeth, so as to make it accessible to the operation of filling, removes the decay. True, in the mouths of the young, such teeth can be often advantageously separated by the use of slips of India rubber, or what is far better, wedges of soft wood; yet even then the edges of the enamel forming the outer surfaee of the cavity should be trimmed away, so that the edge or border of the cavity shall be firm, that it may not crumble away after the plug is inserted; and what is equally important is, that two teeth thus plugged may not again come in contact—allowing the plugs to press on other—or even the approximal edge of a tooth to imping on the plug of the other. Plugs should never require any extraneous force or circumstance to retain them in the teeth, but should be left so that by the use of floss silk, or a handkerchief, they can be kept clean.

But we digress. We wish to expose the prejudice against the proper use of the file. In the use of the file on the front teeth, the object should be to remove the disease, at the same time to preserve the front or labial surface of the teeth as perfect as possible. This would then indicate the necessity of cutting most from the inner or palital surface, and this should be done so that the dip of the disease is entirely removed, and no depression left at that point sufficient to retain particles of food, or any extraneous matter. Cutting instruments, as well as the file, are necessary for the proper performance of this operation. In all cases where admissible, the teeth should be so separated that, if they should close together, a shoulder near the neck above the decay will prevent the filed surfaces from coming in contact. Often the disease can be thus removed, and the palital surface left so, that the action of the tongue will prevent the lodgement of extraneous matter. In all cases the scratches of the file should be well removed, and the bone and filed enamel well polished. A well polished hippopotamus tooth will last longer in the mouth than one left rough by the action of the file.

Dental disease, like other disease in general, has an acute stage, which, if left alone, often runs its course in a few years, and either ends in the destruction of all the teeth, or in a chronic stage—which may linger for life and not do as much injury in twenty years, as before, in four or five. It does not, however, always assume this marked appearance,—or at least the acute stage may not end for a longer period, depending upon the pathological condition of the system, diet of the individual, &c., &c.

Indeed, at times, the dental irritation becomes so great that all effort of nature to restore healthy action will utterly fail. How often do we find persons of from 16 to 20 years with ten or twelve teeth slightly decayed. The enamel has just been decomposed, and the disease has commenced its ravages on the bone of the tooth;—from a slight tenderness in one or more of these, they are induced to apply to a dentist. They have no idea so many are effected—his friends tell him his teeth are too much crowded, and advise their separation—the operator urges this also—he consents

—his teeth are separated—no regard is paid to the removal of the disease, or even the manner of their separation—but he is left with the assurance that his teeth will last for life. The fact is, the acute stage of the disease has just set in—the pressure of the teeth, instead of being the cause of the disease, is that which favors the action of the *direct* cause itself,—disease has already commenced. The file has not removed this—and the patient is left as the man who has swallowed poison, who, after it has reached the circulation, takes an emetic and throws up the dregs of that which has not yet reached his vitals—he thinks his stomach free, and therefore must needs get well. Vain hope—the whole body writhes under the poisoned current which fast flows to its last ebb—he dies, not because the stomach has not been rid of the poison, but because when that was relieved it did not necessarily remove the impression which had been made on the system at large.

This kind of treatment has rendered the filing of teeth of very doubtful utility in the minds of the public. They look more at the surface of things than into the minutia. They see the teeth have been separated, and that now in one or two years thereafter, they are lost. The consequence is, the injury is placed to the filing of the teeth. How soon would they have decayed if not filed? This question never enters their thoughts. The fact is, we cannot see any cause why the mere separation of the teeth when decaying should in the least effect the disease already existing;—but we can see ample cause why an imperfect operation of the kind may fail to arrest that disease.

The mere separation of the teeth with the file—not removing the entire decay—only takes away a portion of the tooth already destroyed by disease, but leaves the deep seated cause at work as before. True, portions of the sound and solid bone and enamel may be removed, yet we never find disease seating itself thereafter at these points—except as having its connection with the original decay. We have seen teeth, incisors as well as molars, one-half removed with the file and cutting instruments, remain for years without further decay. We have seen, too, persons of intelligence, who could quote Pope and Byron perhaps better than the bible, yet attribute the loss of their teeth to the use of the file, when only two had been separated, and they were now the best teeth in their mouth—yet they imagined some destructive influence had been exerted by the file, so that it had occasioned the decay of their other teeth. It becomes us as operators to remove this prejudice from the minds of our patients. The success of our operations, whether in filling the teeth or arresting of the decay by the use of the file, very much depends on the separation of the teeth so as to expose the cavity, or the entire removal of the disease as before described.

It would be well to bear in mind that caries of the teeth is not always dark; that decomposed bone often retains its color for some time, but healthy dentine is always hard, and the cutting instrument cuts it but slowly compared to that acted upon by disease. The latter often crumbles under the edge of the instrument like chalk, while the former is hard like steel. An experienced operator, though blind, could tell when his instrument reaches the healthy portion of the tooth.—Cin. Ed.

